# Field-Based High-Throughput Phenotyping for Maize Plant Using 3D LiDAR Point Cloud Generated With a “Phenomobile”

**DOI:** 10.3389/fpls.2019.00554

**Published:** 2019-05-07

**Authors:** Quan Qiu, Na Sun, He Bai, Ning Wang, Zhengqiang Fan, Yanjun Wang, Zhijun Meng, Bin Li, Yue Cong

**Affiliations:** ^1^Beijing Research Center of Intelligent Equipment for Agriculture, Beijing Academy of Agriculture and Forestry Sciences, Beijing, China; ^2^Department of Biosystems and Agricultural Engineering, Oklahoma State University, Stillwater, OK, United States; ^3^College of Mechanical and Electronic Engineering, Agricultural University of Hebei, Baoding, China; ^4^Department of Mechanical and Aerospace Engineering, Oklahoma State University, Stillwater, OK, United States; ^5^College of Mechanical and Electronic Engineering, Northwest A&F University, Yangling, China

**Keywords:** high-throughput phenotyping, 3D LiDAR, mobile robot, maize, point cloud, field-based

## Abstract

With the rapid rising of global population, the demand for improving breeding techniques to greatly increase the worldwide crop production has become more and more urgent. Most researchers believe that the key to new breeding techniques lies in genetic improvement of crops, which leads to a large quantity of phenotyping spots. Unfortunately, current phenotyping solutions are not powerful enough to handle so many spots with satisfying speed and accuracy. As a result, high-throughput phenotyping is drawing more and more attention. In this paper, we propose a new field-based sensing solution to high-throughput phenotyping. We mount a LiDAR (Velodyne HDL64-S3) on a mobile robot, making the robot a “phenomobile.” We develop software for data collection and analysis under Robotic Operating System using open source components and algorithm libraries. Different from conducting phenotyping observations with an in-row and one-by-one manner, our new solution allows the robot to move around the parcel to collect data. Thus, the 3D and 360° view laser scanner can collect phenotyping data for a large plant group at the same time, instead of one by one. Furthermore, no touching interference from the robot would be imposed onto the crops. We conduct experiments for maize plant on two parcels. We implement point cloud merging with landmarks and Iterative Closest Points to cut down the time consumption. We then recognize and compute the morphological phenotyping parameters (row spacing and plant height) of maize plant using depth-band histograms and horizontal point density. We analyze the cloud registration and merging performances, the row spacing detection accuracy, and the single plant height computation accuracy. Experimental results verify the feasibility of the proposed solution.

## Introduction

The explosive growth of global population leads to an urgent demand for the increase of crop production. It is estimated that cereal yield has to keep a yearly increasing rate higher than 2.4%, to double the current yields by 2050 (Tilman et al., 2011; Ray et al., 2012, 2013). However, traditional extensive breeding methods can only bring a yearly increasing rate about 1.3% at extreme (Ray et al., 2012, 2013). To enhance the yield increasing trends, more and more attention is devoted to molecular and genetic technology inspired breeding methods. These methods have promising prospects on dissecting the generating mechanisms of excellent trait characters, such as yield and stress tolerance (Araus and Cairns, 2014; Chen et al., 2014). Excellent characters will be found through screening of massive traits, which are produced by different molecule or gene combinations. As traditional artificial screening suffers from low speed, high workload and poor uniformity, which becomes a bottleneck for advanced breeding, “high-throughput phenotyping” comes to the stage and becomes a hot topic (Araus and Cairns, 2014).

High-throughput phenotyping can help the breeders to select good traits among a large quantity of samples and to conduct a closed-loop observation for phenotype-gene correlations (Furbank and Tester, 2011). Different sensing technologies (Fahlgren et al., 2015; Lin, 2015; Singh et al., 2016) and different sensor carriers (Yang et al., 2014; Haghighattalab et al., 2016; Mueller-Sim et al., 2017; Virlet et al., 2017) have been applied in the field of high-throughput phenotyping.

Camera based sensing technologies constitute most of the popular phenotyping sensors, including colored cameras, hyperspectral cameras, thermal imaging cameras, chlorophyll fluorescence cameras, and RGB-D cameras, etc. In fact, sensors with different spectral bands enjoy different advantages. Through visible light imaging, colored cameras can acquire visible phenotype parameters, such as shape, color, and texture. These parameters can be employed to monitor plant growth (Li et al., 2016), compute germination percentage (Dias et al., 2011), describe root structure (Richard et al., 2015), and induce leaf area index (LAI) (Virlet et al., 2017), etc. Hyperspectral cameras can cover a wide range spectrum between visible and near-infrared regions. They can detect plant stress response (Asaari et al., 2018), leaf chemical properties (Pandey et al., 2017), etc. Thermal imaging cameras can catch the crop emitted radiation information and be applied for LAI measuring (Bolon et al., 2011), drought tolerance monitoring (Tejero et al., 2015), etc. Chlorophyll fluorescence cameras can obtain physiological information of plants, such as net photosynthesis rate, stomatal conductance, transpiration rate, etc. They can be used to detect plant stress, drought stress (Chen et al., 2014) and salinity stress (Awlia et al., 2016) for example. RGB-D cameras can provide both color image and depth image in the same frame. Thus, 3D plant information can be quickly obtained through close range observation with RGB-D cameras, such as plant height (Jiang et al., 2016) and plant structure (Andújar et al., 2016).

LiDAR is another popular sensor for high-throughput phenotyping. It is good at obtaining high-accuracy range information by firing laser beams. Near range and high-accuracy (micrometer up to millimeter level) 2D LiDAR sensor can be used for indoor and single plant phenotyping. A LiDAR sensor can be mounted on the end-effector of a manipulator, which moves the sensor around the plant to generate 3D point clouds (see, e.g., Chaudhury et al., 2015; Kjaer and Ottosen, 2015). Fine phenotyping, such as wheat ear volume calculation (Paulus et al., 2013), can be conducted based on reconstructed 3D models. 2D line-scan LiDAR can also be applied on outdoor crop-row phenotyping, working together with GNSS (Global Navigation Satellite System) receivers. The sensor will be mounted on a moving platform to scan the crop row from a top view. 3D point clouds for different plants in the row will then be generated. Plant height and structure can be obtained (Sun et al., 2018) with a lower accuracy level (centimeter). 2D line-scan LiDAR are also employed for canopy phenotyping (Garrido et al., 2015; Colaço et al., 2018). The sensor is mounted on a moving platform to scan the plant from a side view. The canopy structure related parameters, such as plant height, LAI and canopy volume, can be obtained. Note that although multi-line-scanning 3D LiDAR is popular in autonomous driving for detecting road, obstacles, and pedestrians (Zhou and Tuzel, 2018), few researchers have used it in phenotyping.

Phenotyping sensor carriers are called phenotyping platforms. Different platforms can act as phenotyping platforms, including fixed platforms, flying platforms, and mobile platforms. Fixed platforms mainly appear in indoor phenotyping scenes (Yang et al., 2014; Guo et al., 2016). Plants in flowerpots or plates will be placed on a conveyor belt and transported to different observation spots with different sensors. This type of platforms can give 360-degree panoramic view of plants, which offers the capability to collect the most rich and detailed information. Flying platforms can be airships (Liebisch et al., 2015), helicopters (Chapman et al., 2014), unmanned aerial vehicles (Haghighattalab et al., 2016; Hu et al., 2018), etc. They are the fastest platforms who can cover a large area of farmland within a short time. But they can only conduct observation on top of the canopy. Mobile platforms are the most active research area of high-throughput phenotyping platforms, and they can be categorized into fully-mobile platforms and semi-mobile platforms. Semi-mobile platforms are usually built based on mechanisms similar to Gantry crane (Virlet et al., 2017). Sensors, such as LiDAR, hyperspectral camera, and colored camera, are installed on the crossbeam and move together with Gantry crane on rails. Restricted by the rails, semi-mobile platforms can only cover parts of a parcel. But benefited from the high payload capability of Gantry crane, observation of semi-mobile platforms can fuse rich and various sources of senor information. Fully-mobile platforms can be handcarts (Nakarmi and Tang, 2014), agricultural machineries (Andrade-Sanchez et al., 2014), and mobile robots (Cousins, 2015; Mueller-Sim et al., 2017). Equipped with different sensing payloads, fully-mobile platforms can take in-row and canopy-top observations for multiple parcels in a close and quick manner.

More and more crops have been measured with high-throughput phenotyping solutions, including maize (García-Santillán et al., 2017), sorghum (Bao et al., 2019), soybean (Pandey et al., 2017), wheat (Banerjee et al., 2018), barely (Paulus et al., 2014), cotton (Sun et al., 2018), etc. Different phenotyping parameters can be extracted, such as plant height (Sun et al., 2018), row spacing (García-Santillán et al., 2017), stem width (Fernandez et al., 2017), leaf area and length (Vijayarangan et al., 2018), etc. Among all the parameters, plant height and row spacing are common parameters for field crop phenotyping. A large number of literatures show that plant height and row spacing have strong influences on canopy structure and light attenuation (Maddonni et al., 2001), radiation interception (Tsubo and Walker, 2002), yield (Andrade et al., 2002), nitrogen availability (Barbieri et al., 2000), etc. The main sensing technologies for plant height and row spacing are computer vision and laser scanning. Row spacing is often detected by computer vision through a row-tracking and top-view manner (Zhai et al., 2016). Cameras are mounted on tractors or other moving platforms with a downward pitch angle. As the platforms move along the rows, crop rows can be detected in the camera view sight and row spacing will be computed. This kind of methods can only detect several row spacing values for each run. Plant height can be detected with both top-view (Sun et al., 2018) and side-view (Colaço et al., 2018) manners. A platform can carry a LiDAR/camera sensor and move parallel to the crop rows. Thus, point clouds or 3D models for the plants will be generated one by one and then employed to determine plant heights. The speed of current high-throughput phenotying solutions for plant height and row spacing strongly depends on the platforms’ moving velocity. Parameters are obtained row-by-row or plant-by-plant. Fast phenotyping solution for parcel level plant group has not been reported.

Although a great stride has been made on high-throughput phenotyping, there are still many open questions. Taking quick ground observations for plant groups on a multi-parcel level is one of these questions. Current existing solutions can only take either canopy-top group observation or side-view single-plant observations. Observing a plant group with side-view and obtaining phenotying parameters simultaneously is still challenging. To tackle this problem, we propose a “phenomobile,” a fully-mobile platform based on an agricultural mobile robot (Qiu et al., 2018). We employ “Velodyne HDL 64E-S3” as the main phenotyping sensor, which has been widely used on autonomous vehicles (Navarro et al., 2017). Our solution can carry out side-view and group observations around the parcel, not in-row. This avoids interference from potential collision between the plant and the robot. Our experimental results demonstrate that our “phenomobile” can acquire plant height and row spacing information with a satisfying accuracy and speed.

## Materials and Methods

### Hardware Setup

#### Mobile Platform

We employ “AgriRover-01,” a self-developed agricultural mobile robot for crop information collection, to act as the senor carrier. This robot has six motors, four in-wheel motors for driving and two motors for steering. The four driving wheels are grouped into two pairs: front pair and rear pair. For each pair, we use a steering gear to make them turning simultaneously. An extended Ackerman Steering Principle is implemented to carry out the coordinated movement control. For details, please refer to the reference (Qiu et al., 2018).

#### Sensor Setup

We employ a 3D LiDAR, Velodyne HDL64E-S3, as the main phenotyping sensor. This sensor has 64 horizontal scan lines of 360 degrees, with a vertical field of view of 26.8 degrees and range accuracy of 2 cm. Thus, it can easily acquire high-resolution 3D point clouds of crops. Table 1 lists key sensor parameters selected from the Velodyne HDL64E-S3 manual.

**Table 1:** Key parameters of Velodyne HDL64E-S3.

Key parameters	Values
LiDAR class	64 laser lines/detectors
Field of view/scanning angle (azimuth)	360 degrees
Vertical field of view	26.8 degrees
Angular resolution (azimuth)	0.09 degrees
Accuracy (one sigma)	<2 cm distance accuracy
Operation range (distance)	120 m range for cars and foliage


**FIGURE 1:**
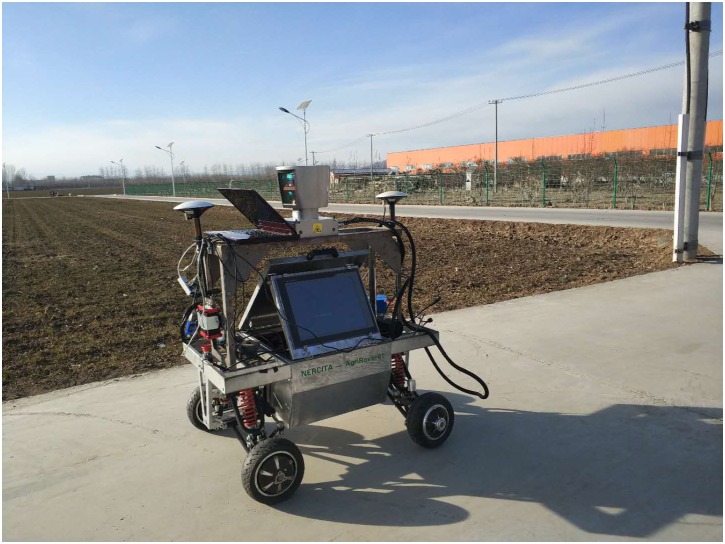
Velodyne HDL64E-S3 on the robot.

We install the LiDAR on a self-designed bracket on top of the mobile robot as shown in Figure 1, making the sensor’s center of gravity about 0.9 m above the ground. The localization information is obtained through a GNSS receiver set, which has two antennas. An industrial personal computer collects the localization signals through RS485. A notebook computer collects the LiDAR senor outputs through Ethernet. On the notebook computer, Robotic Operating System (ROS)-indigo is installed on Linux (Ubuntu 14.04). The Velodyne data acquisition code is downloaded from GitHub^1^, whose author is Jack O’Quin from Austin Robot Technology. As the original code is not developed for Velodyne HDL64E-S3, we make some modifications to one of its files^2^ to match the new features of S3. First, line 37 is changed to

(1) private_nh.param (“model”,config_.model, std :: string(“64E_S3”));

second, a short paragraph is added to line 46 as

(2) $$\begin{array}{l} else\;if\;(config\_.model=="64E\_S3")\\ //\mathrm{generate}\;13340\;\mathrm{per second, where 1333440}\\ \mathrm{// = 4167 (per second) * 64 * 5}\\ \{\;\mathrm{//1 package holds 384 points}\\ \mathrm{// (12 fire/packet and 32 laser/fire)}\\ \mathrm{packet\_rate = 3472}\mathrm{.5;}\;\mathrm{//1333440/384 = 3472}\mathrm{.5}\\ \mathrm{model\_full\_name = std :: string ("HDL-") + config\_}\mathrm{.mode;}\\ \} \end{array}$$

### Preliminary Information of the Experiments

#### Avoiding the LiDAR Blind Area

As the vertical field of view of the LiDAR ranges from -24.8° to +2°, there exists a blind area. To eliminate the influence of blind area, we choose to scan the plant at a distance larger than the diameter of the blind area. We compute the diameter of the blind area *D* as

(3) $$D=\frac{H+h}{\tan\;\alpha }$$

where *H* is the height of the bracket on the mobile robot “AgriRover-01,” *h* is the distance from the origin of the LiDAR’s coordinate to its base, and α is the maximum depression angle of the sensor (-24.8°). Here, we set *H* as 0.91 m and *h* as 0.25 m according to manual measurements. Then, we obtain *D* as 2.49 m.

#### Landmarks

**FIGURE 2:**
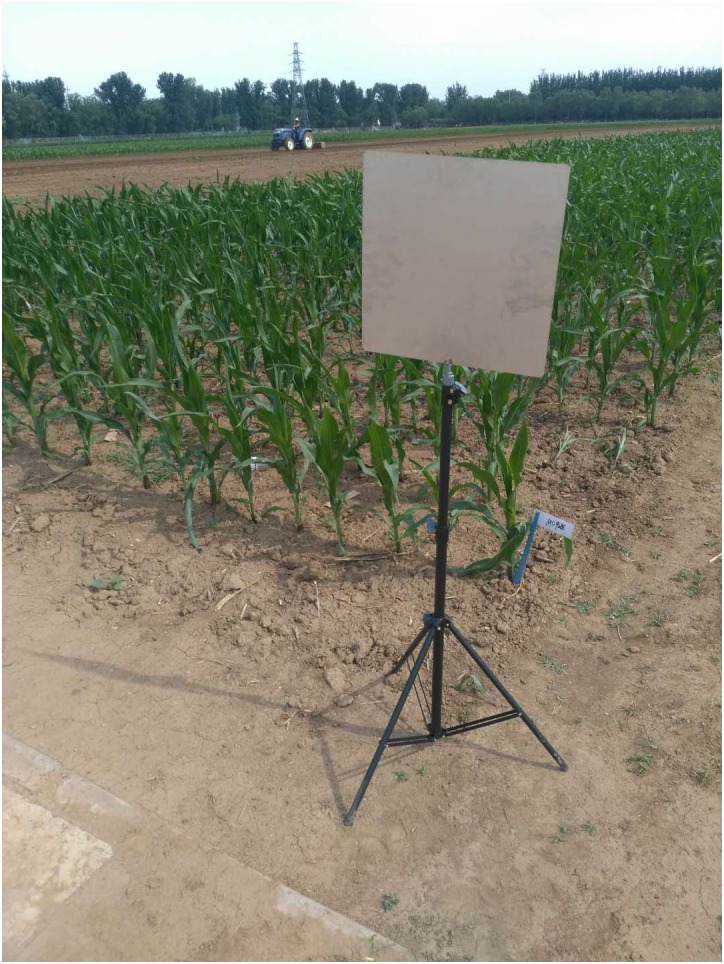
Landmark in the experimental field.

We choose a multi-view solution to increase the point density and compensate for the self-occlusion of plants. As a result, the point clouds from different observation spots need to be merged into one. Point cloud matching is a challenging task and requires large quantities of computation resources. To simplify and speed up the point cloud merging process, we set several landmarks in the experimental field, parallel to the moving direction of the robot. The landmarks are 40 cm × 30 cm rectangle plates, which are made of stainless iron and stand on tripods. Figure 2 shows one of the landmarks in the experimental field.

#### Experimental Settings

**FIGURE 3:**
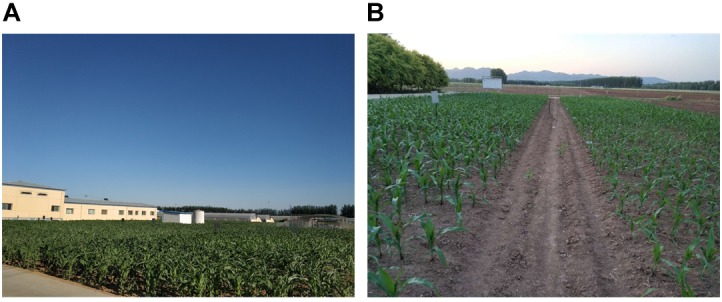
Two experimental parcels. **(A)** Is parcel-1; **(B)** is parcel-2.

We choose two parcels as our experimental fields. Parcel-1 is 40 m × 30 m and parcel-2 is 33 m × 17 m. For both parcels, the robot moves on the cement floor beside the parcels. For parcel-1, floor-1 is 0.2 m higher than the farmland. For parcel-2, floor-2 is a small slope, whose top and bottom are 0.45 and 0.1 m higher than the farmland, respectively. Also, there is a corridor crossing through the middle area of parcel-2. In parcel-1, the height of the maize plants is around 0.7 m, 30 days after sowed. In parcel-2, the height of the maize plants is around 0.6 m, 20 days after sowed. Figure 3 shows the two parcels.

**FIGURE 4:**
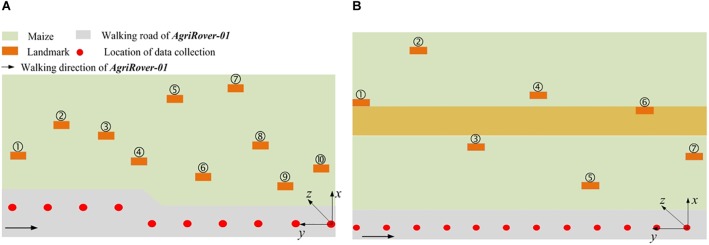
Experimental layouts of parcel-1 and parcel-2. **(A)** Is parcel-1; **(B)** is parcel-2.

During the data collection process, the robot moves along the cement floor beside the parcel and carries out the task in a stop-and-go manner. When the robot stops, the LiDAR performs several scans with a preset frequency of 5 Hz. For each scan, a 360° point cloud with more than 1.33 million points is generated and saved. The two adjacent observation spots are about 1 m apart from each other. For each spot, the robot stays for 10 s at least to collect enough scan data. Data from different observation spots are merged by post-processing. We deploy the landmarks at random positions, with the lower borders at least 0.1 m higher than the plants. Figure 4 shows the experimental layouts of the two different parcels. The green area stands for the maize field. The gray area stands for the cement floor. The orange rectangle stands for the landmark. The red circle stands for the data collection spot. The arrow points out the moving direction of AgRover-01. The coordinate system for each parcel scenario is marked in Figure 4. We select the origin of the LiDAR’s coordinate system at the last observation spot as the origin of the global coordinate system for each scenario. We define the direction facing to the maize field as the positive direction of X coordinate, the left side direction as the positive direction of Y coordinate, and the upward direction as the positive direction of Z direction.

### Data Processing

#### General Framework of Data Processing

**FIGURE 5:**
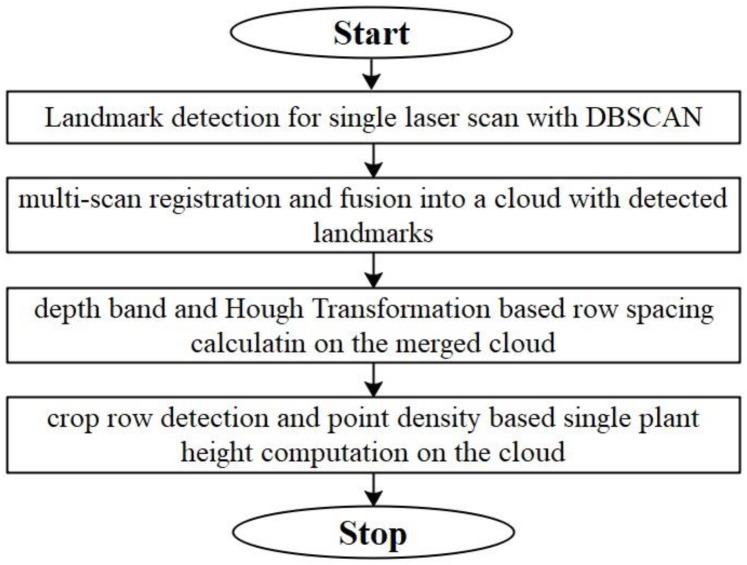
Flowchart for the general framework of data processing.

The general framework of data processing contains four steps: (1) DBSCAN based landmark detection in a single scan; (2) SAC-IA and Iterative Closest Point (ICP) based cloud registration and fusion; (3) row spacing calculation based on depth-band and point density histogram; (4) single plant height computation based on detected row and point density. Figure 5 shows the flowchart for the whole data processing framework. To speed up the coding process, we develop the data processing project using Point Cloud Library (Rusu and Cousins, 2011).

#### ROI Abstracting

ROI (region of interest) abstracting is a very important preliminary processing step. It helps to cut down the data computation load. As the distance range of our LiDAR is 120 m and the horizontal field of view is 360 degrees, each sensor frame covers an annular area with an external diameter of 120 m and an internal diameter of 2.49 m (the diameter of the blind area *D*). To abstract the ROI on the horizontal plane, we first cut off the half annular opposite to the parcel. Then, we select the ROI for each observation spot using manually chosen horizontal thresholds. To further eliminate the noise coming from the uneven terrain, we delete the bottom part of the point cloud with a threshold of 0.005 m. That is, we first find the minimum height value *z*_min_ and then remove all the points with their height values lying between [*z*_min_, *z*_min_ + 0.005].

#### Landmark Detection

After abstracting the ROI, we employ a multi-view solution to tackle the self-occlusion problem and to increase the cloud point density. When merging point clouds coming from different observation spots, we use landmarks to decrease the computation load and speed up the matching process. Thus, detecting the landmarks is the key point. We first detect a corner point for each landmark, then generate a virtual point cloud for each landmark through interpolation, and finally obtain the translation matrix between two observation point clouds by registering the two corresponding virtual landmark point clouds.

**FIGURE 6:**
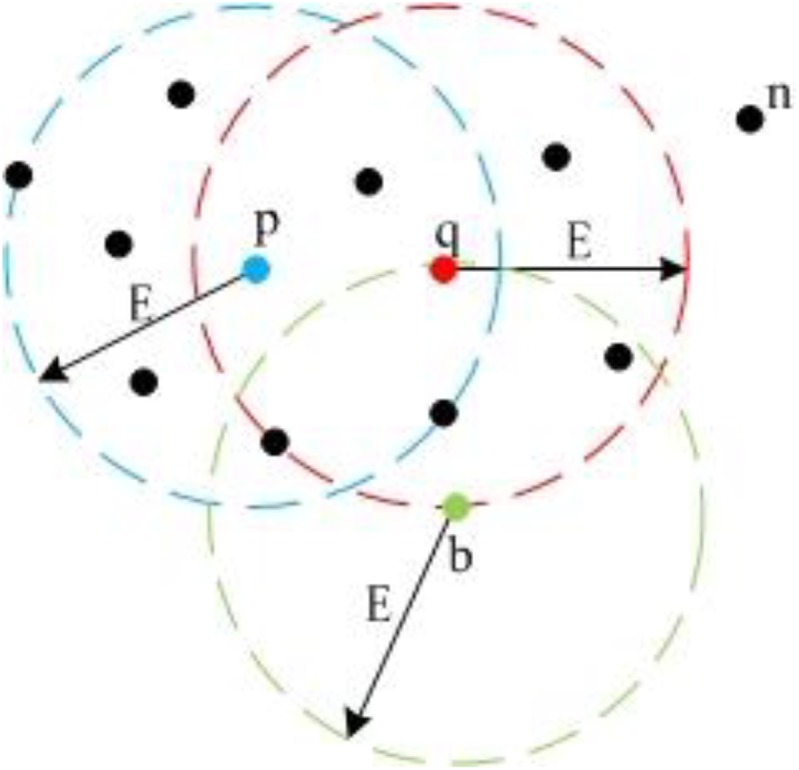
Different point types in DBSCAN. Because **p** and **q** both have more than five neighbors within *E* circle adjacent area, they are Core Points; **b** has less than five neighbors, but it lies in the adjacent area of **q**, so **b** is a Border Point; **n** is a Noise Point.

We employ DBSCAN (Ester et al., 1996; Huang et al., 2017) to detect the landmark point clouds. Based on a preset minimum point number (*MinPts*) within the adjacent region of one point, DBSCAN categorizes points into three types: “Core Point,” “Border Point,” and “Noise Point.” Core Points have more points than *MinPts* within their adjacent region. Border Points have fewer points than *MinPts* within their adjacent region, but they lie in the adjacent region of one or more Core Points. Noise Points have fewer points than *MinPts* within their adjacent region and do not lie in the adjacent region of any Core Points. Figure 6 gives an example for the definition of the three point-types.

**FIGURE 7:**
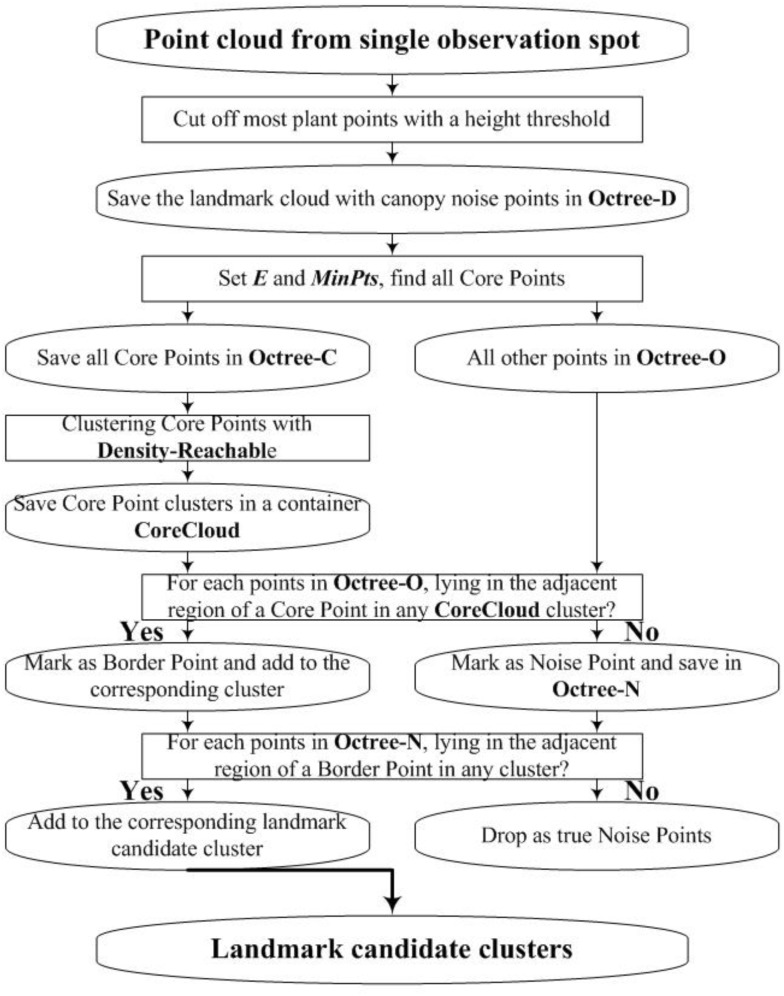
Flowchart for DBSCAN and Octree based landmark detection.

In Figure 6, we set *MinPts* as 5. Because point **p** and **q** both have six points in their adjacent region, they are Core Points. Because point **b** does not have five points in its adjacent region, it is not a Core Point. However, **b** is in the adjacent region of **q**. Thus, it is a Border Point. Point **n** does not have five points in its adjacent region and is not in the adjacent regions of **p** or **q**, so **n** is a Noise Point. Also, Figure 6 demonstrates the concept of “Density-Reachable,” which helps to merge over-segmented clusters. In Figure 6, *E* is the diameter of the preset adjacent region. The distance between **p** and **q** is smaller than *E*, meaning that **p** and **q** both lie in each other’s adjacent region. For this situation, we call **p** is Density-Reachable for **q** (or **q** is Density-Reachable for **p**). If **p** and **q** belong to different clusters because of over segmentation, we can merge the two small clusters into one big cluster. Figure 7 shows the flowchart for landmark detection. Note that we use Octree (Wurm et al., 2010) to organize the rough point cloud in order to speed up the point searching operation.

After the landmark candidate clusters are detected, we first delete the canopy noise clusters with a minimum cluster height *ch*. Here, we set *ch* as 0.1 m. Then we determine one upper corner point and generate a virtual landmark point cloud. There are many approaches to determining the upper corner of the landmark, such as setting Z coordinate value as the biggest Z value of the cloud, Y coordinate value as the smallest Y value, and X coordinate value as the mean of all X values. To generate the virtual landmark point cloud from the corner point, we assume that the landmark plane is perpendicular to the field terrain and interpolate points with preset vertical and horizontal steps.

#### Cloud Fusion by Registering the Corresponding Virtual Landmarks

**FIGURE 8:**
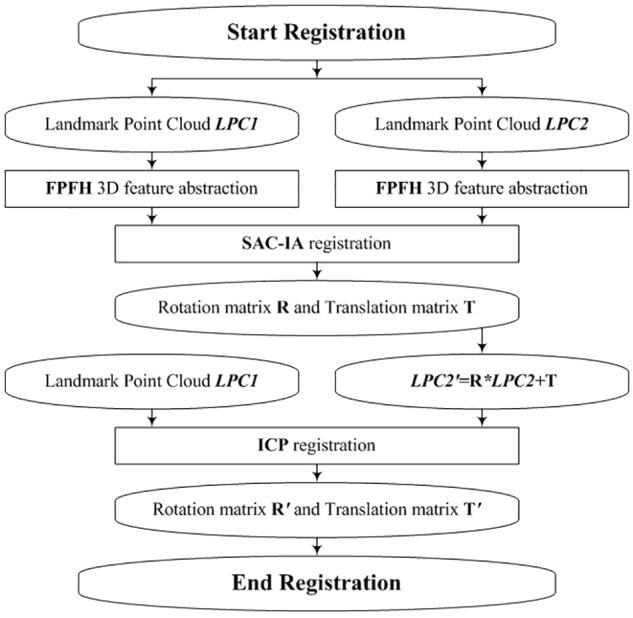
Flowchart for virtual-landmark-based registration.

Our registration process consists of two steps: rough registration with Fast Point Feature Histograms (FPFH) and SAmple Consensus Initial Alignment, SAC-IA (Rusu et al., 2009) and precise registration with ICPs (Besl and Mckay, 1992). The rough registration is used to avoid the local minimum problem of ICP. Figure 8 shows the flowchart of the virtual-landmark-based registration process. The obtained rotation matrix **R′** and translation matrix **T′** can be employed to fuse two corresponding observation point clouds.

#### Row Spacing Calculation

**FIGURE 9:**
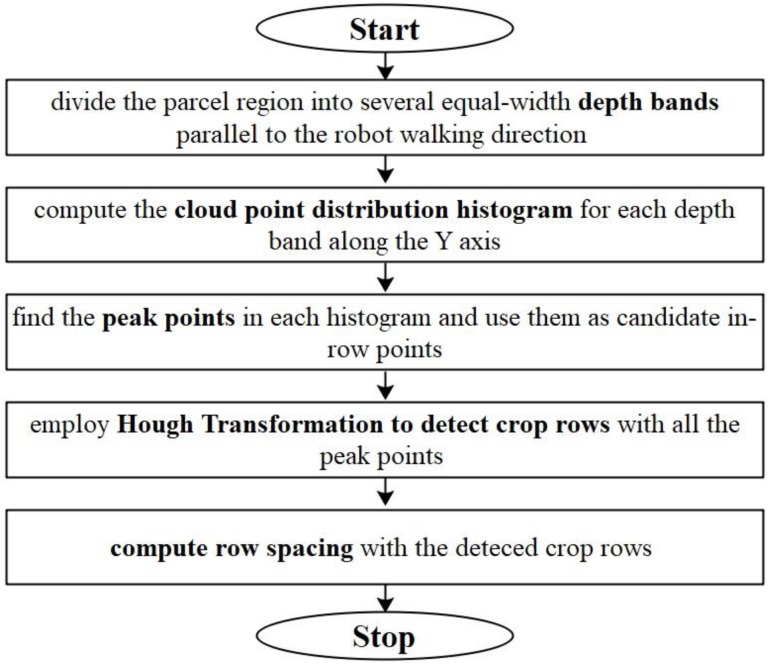
Flowchart for row spacing computation.

Before the row spacing calculation, we project all the cloud points within the ROI onto a horizontal plane. We compute row spacing values with the following five steps: (a) divide the parcel region (or the ROI) into several depth-bands with equal band width on the horizontal plane and keep the depth-bands parallel to the moving direction of the robot; (b) compute the cloud point distribution histogram of each band along the Y axis (for the details of the definition of Y axis, please refer to Figure 4); (c) find the peaks in the band point histograms. We assume that the stem part of a maize plant should produce more laser scan points than the leaf parts, if all the points are projected on the horizontal plane. Thus, we choose the peaks as candidates of in-row points; (d) employ Hough Transformation (Duda and Hart, 1972) to detect the crop rows. Here, we set an angle searching range of [75°, 105°] as the crop rows are assumed nearly perpendicular to the robot moving direction. Also, a minimum point limit of 3 is set to the Hough Transformation. (e) Compute the row spacing (*RS*) values as the nearest distances between two adjacent crop rows. The flowchart for row spacing computation is shown in Figure 9.

#### Plant Height Computation

**FIGURE 10:**
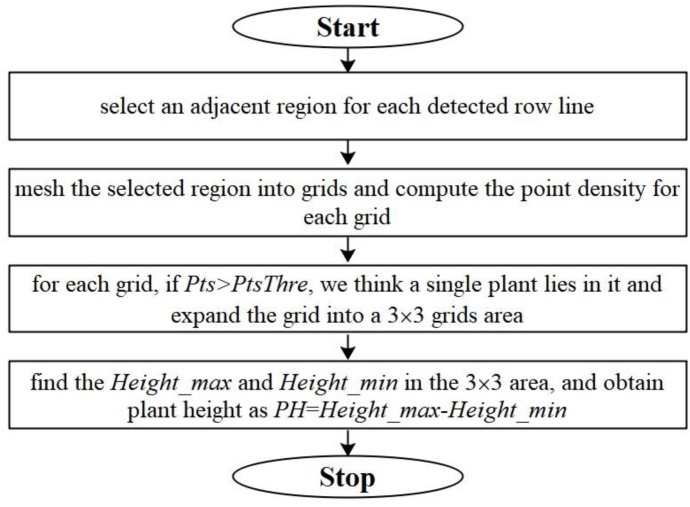
Flowchart for plant height computation.

We detect single plant and compute plant heights for Parcel-2 with the following steps: (a) select an adjacent region for each detected crop row such that the selected region contains the sub-point-cloud of the corresponding row; (b) mesh the selected region with a selected border length (*BL*) on the horizontal plane and compute the point density (*Pts*) in each grid; (c) check the *Pts* for each grid, if *Pts* > *PtsThre* (a preset threshold), we assume that this grid is located around a single plant and expand the grid to a 3 × 3 grid neighborhood; (d) search for the maximum height values of the 3D points within this neighborhood, and set the plant height *PH* as *Height*_max_ – *Height*_min_. Here, *Height*_max_ is the largest value along Z axis of the point cloud located within the 3 × 3 grid neighborhood. *Height*_min_ is a preset height value, which equals to *z*_min_ + 0.005 as in Section “ROI Abstracting.” The flowchart for plant height computation is shown in Figure 10.

### Manual Sampling

**FIGURE 11:**
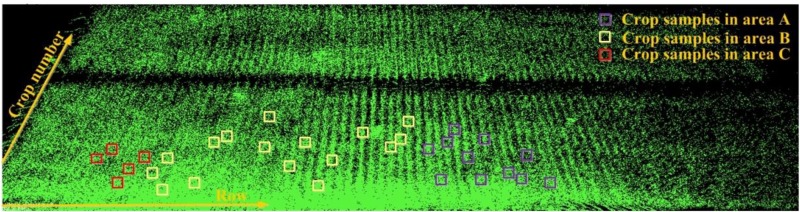
Locations of all manually measured plant height samples.

We conduct manual sampling for row spacing and plant height in Parcel-2. For Parcel-2, each row is approximately perpendicular to the moving direction of the robot. We choose the first row from the parcel ridge at the robot’s start point side, as the first row for row spacing sampling. We manually sample 57 row spacing values one by one from the first row. For plant height, we randomly sample 33 plants. Here, we roughly divide the Parcel-2 area into three sub-areas: area A, area B, and area C, mainly according to the point density. In each row, the plant nearest to the robot is counted as crop No. 1. The locations of all plant height samples are shown in Figure 11. We express the location of each plant with its row number and crop serial number in row as (*row_num*, *crop_num*). In this case, we can find each plant’s corresponding point cloud easily by referring to its row and crop serial numbers.

#### Manual Measurements for Row Spacing

**FIGURE 12:**
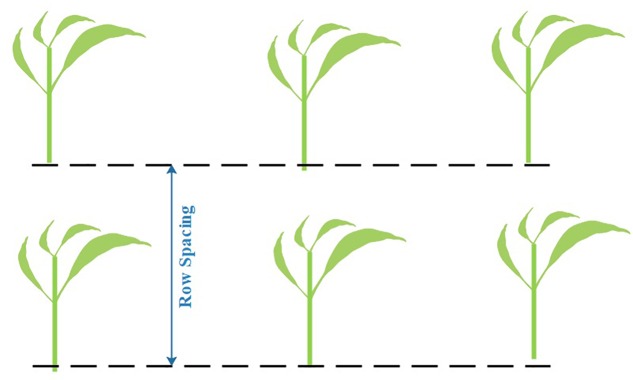
Manual sampling method for row spacing. The dash lines are two adjacent local row-lines.

We use two adjacent plants in the same row to determine a local row-line. The distance between two local row-lines is then measured as the row spacing value (see Figure 12). To reduce the measurement error, we repeat the measurement in three different spots for each row spacing sampling and use the mean value as the final row spacing measurement result.

#### Manual Measurements for Plant Height

**FIGURE 13:**
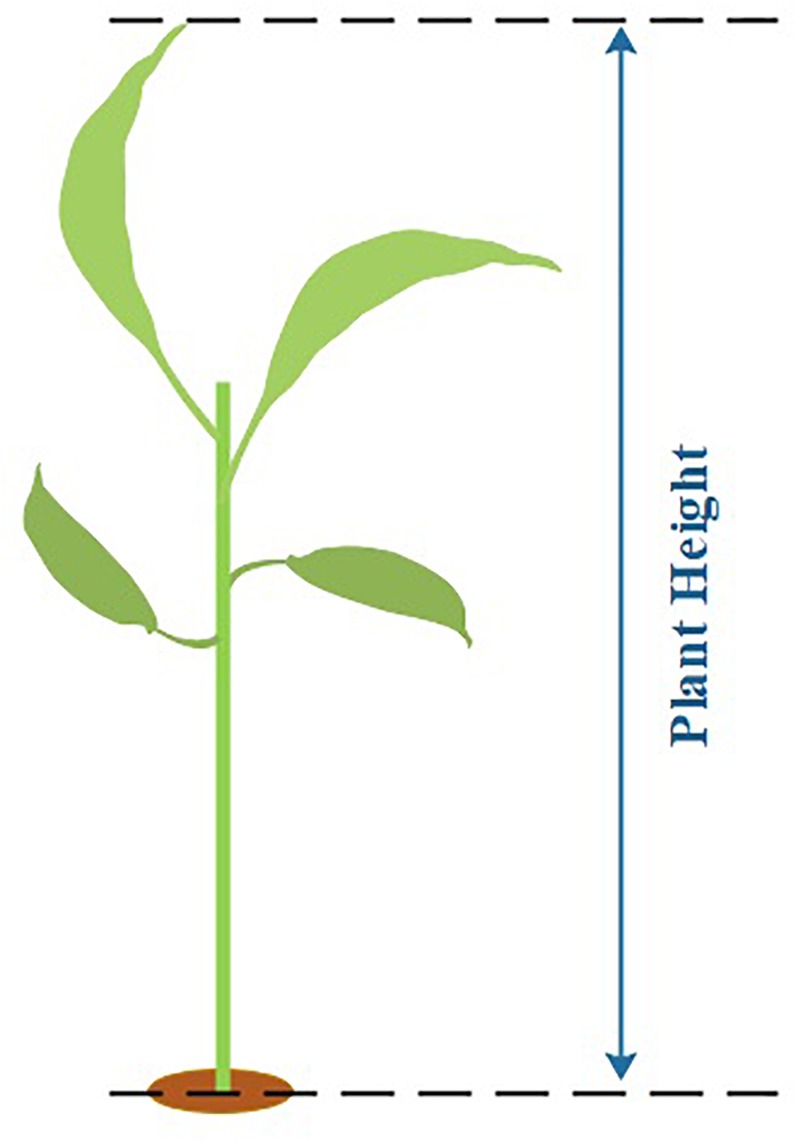
Manual sampling method for plant height.

We assume plant height as the distance from the highest point to the lowest point above ground of the plant (see Figure 13). To reduce the measurement error, we also repeat the measurement three times for each plant sample and use the mean value as the final plant height measurement result.

## Results

### Results of Landmark Detection

We set *E* = 0.52 m and *MinPts* = 5. Here, *E* = 0.52 m is the diagonal length of the landmark plate and 5 is the minimum integer that is larger than 3 (3 dimensions) + 1. We number the observation plot from the start plot of each parcel. With an incremental step of 3 (the reason for choosing 3 is given in Section “Results of Point Cloud Fusion”), we conduct landmark detection for a scan at each selected observation plot. In each parcel, we choose 10 observation plots. For parcel-1, the plots are 4, 7, 10,…, 31. For parcel-2, the plots are 3, 6, 9,…, 30. Four parameters, including Number of Landmarks in View (NLV), Detected Number of Landmarks (DNL), Detection Success Rate (DSR), and Detection Time Duration (DTD), are listed in Tables 2, 3, corresponding to Parcel-1 and Parcel-2.

**Table 2:** Landmark detection results for Parcel-1.

Plot number	4	7	10	13	16	19	22	25	28	31
**NLV**	4	4	4	5	5	6	8	5	5	5
**DNL**	3	3	3	4	4	4	6	4	5	4
**DSR**	75%	75%	75%	80%	80%	66.7%	75%	80%	100%	80%
**DTD (ms)**	401	656	1566	2691	954	401	386	1188	513	1156


**Table 3:** Landmark detection results for Parcel-2.

Plot number	3	6	9	12	15	18	21	24	27	30
**NLV**	5	5	5	6	4	5	5	4	4	3
**DNL**	3	4	4	4	3	4	3	3	3	3
**DSR**	60%	80%	80%	66.7%	75%	80%	60%	75%	75%	100%
**DTD (ms)**	435	649	414	597	898	752	425	398	477	674


From Tables 2, 3, we can see that DBSCAN works quite well. For each observation scan, at least three landmarks can be detected, which strongly supports the cloud registration process. Also, the detection success rate is consistently above 60%.

**FIGURE 14:**
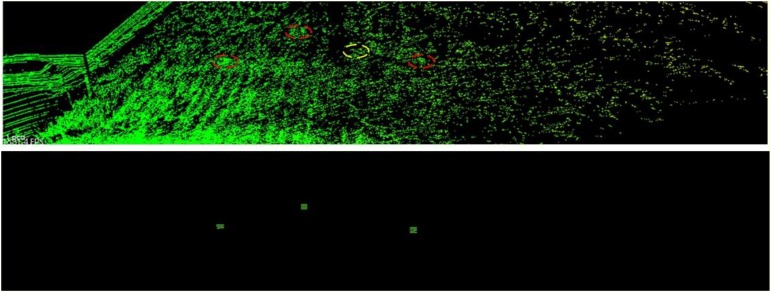
Landmark detection result for plot 2 in Parcel-1.

Figure 14 shows the landmark detection performances at observation plot 2 of Parcel-1. The red dash ellipses indicate the successfully detected landmarks and the yellow dash ellipses indicate the undetected landmarks. The lower part is the generated virtual landmark point clouds.

### Results of Point Cloud Fusion

#### Merging Plot Step Selection

**FIGURE 15:**
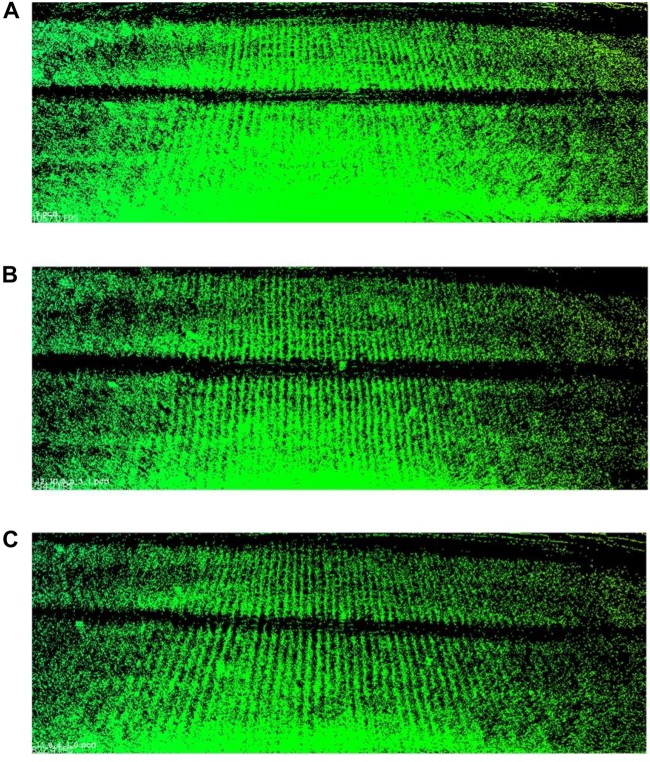
Merged clouds for three different steps. **(A)** Is the merged cloud for “step = 1”; **(B)** is the merged cloud for “step = 2”; **(C)** is the merged cloud for “step = 3.”

We take the laser scans at a series of plots with around 1 m spacing. The more plots we choose to merge, the higher point density we can have and the more registration error we introduce in. We have to balance between point density (information quantity) and error influences. Here, we carry out three merging results for Parcel-2 with different plot incremental steps: all plots (step = 1), half of the plots (step = 2), and one-third of the plots (step = 3). “Step = 2” means we choose a series of plots with around 2 m spacing and merge the scan coming from them. An example plot set for “Step = 2” can be plot 2, 4, 6,…, 30. Table 4 shows the number of the merged scan frames, the point density of merged clouds, and the total registration time for three different steps. Figure 15 shows the merged clouds for three different steps.

**Table 4:** Registration performances of three different steps.

Step	Number of merged scan frames	Point density	Total registration time (ms)
1	30	8,006,400	2.97558e+06
2	15	4,003,200	1.27932e+06
3	10	2,668,800	746,194


From Table 4, we can see that the registration time consumption increases together with the cloud point density. From Figure 15, we observe that case “step = 1” introduce in a lot of registration error, which blurs the point cloud and makes crop rows hard to detect. Case “step = 2” is better but cloud blur is still heavy in the middle region of the parcel. Although case “step = 3” contains less information (low point density), the cloud blur is not significant. Based on the above analysis, we make a compromise between information quantity and registration error influences, by choosing the cloud merging step as 3.

#### Point Cloud Registration and Merging

**FIGURE 16:**
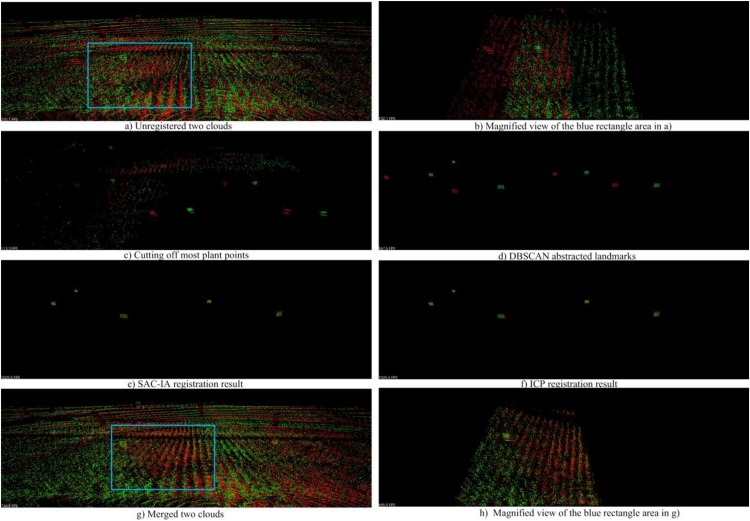
Cloud registration and merging process. **(A)** Shows the unregistered two point clouds; **(C)** shows the landmark points and noise points after cutting off most plant points; **(D)** shows the virtual landmarks generated after DBSCAN; **(E)** shows the SAC-IA registration result; **(F)** shows the IPC registration result; **(G)** shows the merged two clouds; **(B)** and **(H)** show the magnified views of the blue rectangle areas in **(A)** and **(G)**, respectively.

Figure 16 shows an example for the registration and merging process, in which the point clouds are collected from parcel-2. We can see in Figure 16A) that the two unregistered point clouds have obvious drifts. In Figure 16C), we use a height threshold to cut off most plant points. As a result, landmark points and some noise points are kept. Then we employ DBSCAN to detect the landmark point clouds and generate virtual landmarks, as shown in Figure 16D). We can see there are big translation deviations and small rotation deviations between the two original point clouds. In the following step, we carry out rough registration with SAC-IA. The translation deviations are greatly reduced as shown in Figure 16E). We further conduct precise registration with ICP. Note that the registration error is indeed reduced, although we can hardly find obvious improvements in Figure 16F). Based on the registration results, we merge the two clouds and obtain a new cloud with higher point density, shown in Figure 16G). Figure 16B,H are the magnified views of the blue rectangle areas in Figure 16A,G, respectively. From these two magnified views, we can recognize the registration and merging influences clearly.

### Results of Row Spacing Computing

**FIGURE 17:**
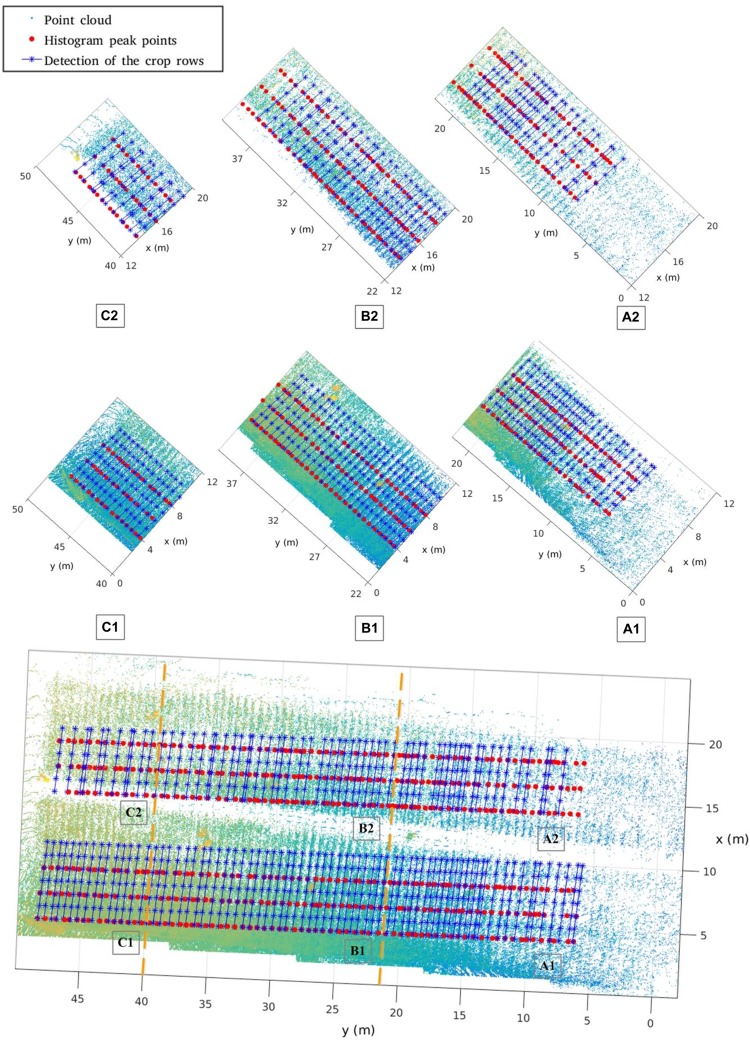
Row detection result for Parcel-2.

Based on the merged point clouds, we carry out the crop row spacing computation for Parcel-2. The row detection result is shown in Figure 17. Here, we also compute 57 row spacing values starting from the same first row as in Section “Manual Sampling.” Thus, each manually sampled row spacing value can be easily identified in the merged Parcel-2 point cloud by detecting the ridge.

In Figure 17, we divide the whole Parcel-2 area into six sub-areas: A1, A2, B1, B2, C1, C2. The Y axis regions for A, B, C sub-areas are (0, 22 m), (22, 40 m), (40, 50 m), respectively. The corridor further separates A, B, C into A1, A2, B1, B2, C1, C2, respectively. Because of occlusion and different laser beam angles, sub-areas have different point densities and information adequacy level. We draw the point cloud, the histogram peaks of depth bands, and the crop rows detected by Hough Transformation, which shows the different row detecting performances in different sub-areas.

**FIGURE 18:**
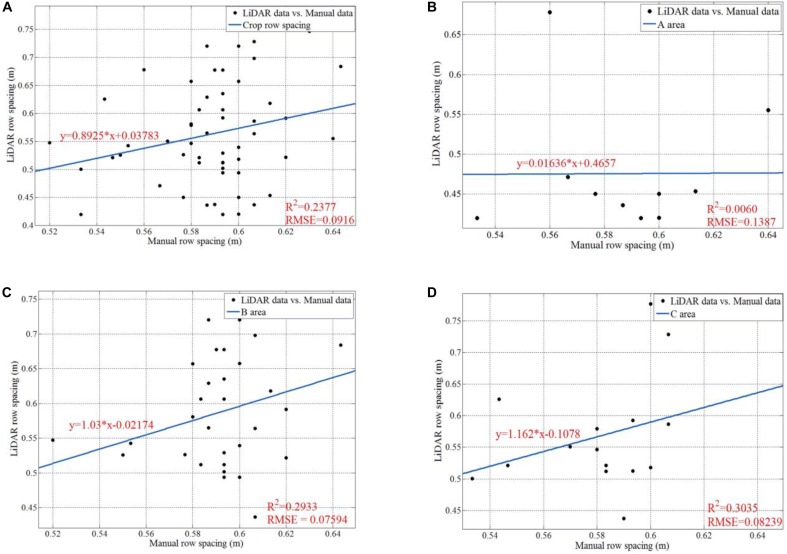
Data analysis for manually sampled and calculated results of row spacing. **(A)** Is the regression line, the R square, and the RMSE for all the 57 row spacing values; **(B)** is the regression line, the R square, and the RMSE for the row spacing values in Area A; **(C)** is the regression line, the R square, and the RMSE for the row spacing values in Area B; **(D)** is the regression line, the R square, and the RMSE for the row spacing values in Area C.

Figure 18 shows the data analysis results for manually sampled and calculated row spacing values. For the whole parcel, the R Square and RMSE of 57 row spacing values are 0.2377 and 0.0916, respectively. We also give analysis results for subarea A, B, and C. We can easily infer that area B (B1 + B2) has the highest row spacing accuracy, which has the smallest RMSE as 0.07594. Although our solution gives lower raw spacing accuracy than other method with mean error of 1.6 cm (Nakarmi and Tang, 2014), it enjoys much higher measurement speed. Our solution can give more than 50 row spacing values in the parcel level for a run, while others can only work in a one-by-one style. Furthermore, the main purpose of this paper is to verify the feasibility of the proposed platform. Thus, we focus on ensuring the entire system and software pipeline working properly. In future work, we will investigate methods to improve the accuracy. We believe that we can significantly improve the accuracy by adding new peak detection algorithms and filtering tools.

### Results of Plant Height Computing

**FIGURE 19:**
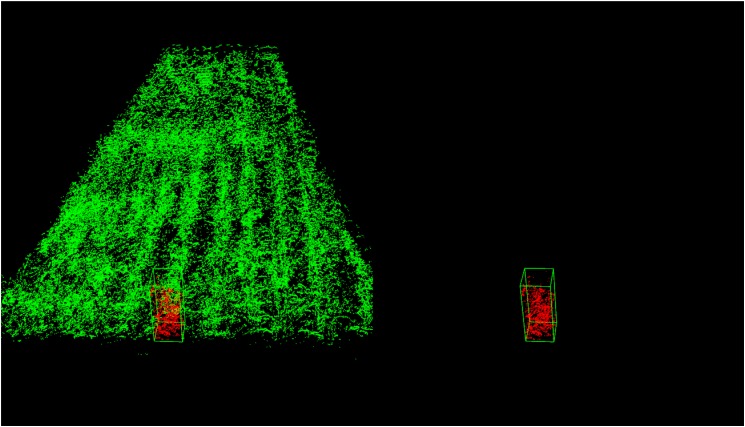
Single plant detection for parcel-1. The detected single plant cloud is in red and marked out with a green bounding box.

Figure 19 shows an example for single plant detection in the plant height computation process from the data of parcel-1.

To verify the feasibility of our solution for plant height computing, we manually measure 33 single plants in Parcel-2 (see Figure 11). Then, we try to find the corresponding plant height values in the point cloud and compare them with the manual measurements. Here, we set *BL* as 0.1 m, which means that the grid size is 0.1 m × 0.1 m. *PtsThre* is determined according to the X axis value: *PtsThre* = 80 for 0 m ≤*x* ≤ 5 m; *PtsThre* = 30 for 5 m < *x* ≤ 8 m; *PtsThre* = 10 for *x >* 8 m.

Tables 5–7 shows the plant height computation results for sub-area A1, B1, C1, respectively.

**Table 5:** Plant height computation results for sub-area A1.

Location (row_num, crop_num)	Manual measurements (m)	Computation results (m)	Errors (m)	Error ratio (%)	x range	RMSE
(58, 3)	0.510	0.651	0.141	27.575	0 ≤ x < 5	0.178
(48, 4)	0.530	0.628	0.098	18.442		
(52, 4)	0.600	0.333	0.267	44.418		
(56, 4)	0.580	0.308	0.272	46.941		
(55, 5)	0.600	0.581	0.019	3.088		
(51, 7)	0.600	0.415	0.185	30.816		
(57, 7)	0.600	0.507	0.093	15.575		
(47, 9)	0.500	0.309	0.191	38.274		
(49, 13)	0.500	0.313	0.187	37.388	5 ≤ x < 8	0.196
(53, 15)	0.510	0.590	0.080	15.693		
(50, 19)	0.600	0.328	0.272	45.391		


**Table 6:** Plant height computation results for sub-area B1.

Location (row_num, crop_num)	Manual measurements (m)	Computation results (m)	Errors (m)	Error ratio (%)	x range	RMSE
(17, 1)	0.510	0.503	0.007	1.352	0 ≤ x < 5	0.058
(36, 2)	0.520	0.609	0.089	17.032		
(21, 3)	0.550	0.495	0.055	10.020		
(15, 5)	0.560	0.621	0.061	10.949		
(32, 6)	0.560	0.572	0.012	2.212		
(16, 7)	0.560	0.593	0.033	5.974		
(37, 8)	0.520	0.611	0.091	17.590		
(29, 10)	0.560	0.616	0.056	9.964		
(43, 11)	0.530	0.673	0.143	26.935	5 ≤ x < 8	0.172
(33, 12)	0.560	0.312	0.248	44.360		
(23, 13)	0.620	0.408	0.212	34.244		
(34, 14)	0.540	0.477	0.063	11.660		
(44, 15)	0.550	0.613	0.063	11.435		
(24, 16)	0.560	0.337	0.223	39.759		
(40, 18)	0.440	0.594	0.154	34.915		
(45, 21)	0.560	0.561	0.001	0.243	x ≥ 8	0.128
(28, 23)	0.500	0.682	0.182	36.317		


**Table 7:** Plant height computation results for sub-area C1.

Location (row_num, crop_num)	Manual measurements (m)	Computation results (m)	Errors (m)	Error ratio (%)	x range	RMSE
(12, 3)	0.450	0.542	0.092	20.415	0 ≤ x < 5	0.097
(13, 6)	0.510	0.573	0.063	12.423		
(9, 7)	0.400	0.521	0.121	30.268		
(14, 8)	0.560	0.575	0.015	2.603		
(10, 9)	0.400	0.542	0.142	35.518		


From Tables 5–7, we see that the plant height computation performances of our strategy are affected by point density and depth value. The part with depth value 0 ≤ x < 5 in sub-area B1 is observed from all 10 plots and suffers from fewest occlusions. Then, we can obtain more accurate plant height values of the plants lying in B1, with *RMSE* = 0.058 m. Comparing with other method with *RMSE* = 0.035 m (Madec et al., 2017), our method can give acceptable plant height accuracy in areas with proper observation conditions. U;

## Discussion

In this paper, we present a field-based high-throughput phenotyping solution for maize, using a 3D LiDAR placed on a mobile robot platform. With the proposed solution, we can obtain the row spacing and plant height information for a parcel level plant group in a run. Each row spacing and each plant height can be estimated by this solution. Also, the robot does not move in-row and thus avoids touching interference with the crop. Experimental results show that our solution can get row spacing and plant height with satisfying speed and accuracy.

From the experimental results, we conclude that the row spacing and plant height calculation performance has a strong relationship with the cloud point density and the cloud registration error. Sparse point density may be caused by occlusion, growing distance, and insufficient observations. To improve the phenotyping accuracy, we plan to collect data around the parcel with proper observation-plot distance. We also plan to improve the cloud registration algorithm to reduce the cloud merging error. As a result, an observation selection strategy and a new cloud registration algorithm will be the main research topic in the future.

Furthermore, we believe that our phenotyping solution can be improved in the following aspects. First, our landmarks are not big enough, which brought in errors and difficulties during the landmark detection process. We may redesign bigger and sturdy landmarks to decrease the influences of strong wind and reduce the detection error, which in turn reduces cloud registration error. Second, we use many preset thresholds, which makes our solution suffer from low robustness and adaptability. In the future, we will investigate the use of more implicit and common features in the field-based maize point cloud, rather than preset thresholds. Third, our solution is a post-processing solution. We will carry out an on-line version soon. Fourth, terrain situation is another key factor for our strategy. For most open field scenarios, the robot will not move on a cement floor. How to choose a proper route and navigate the robot to follow it is an important problem, because it will help provide stable observations. Fifth, only row spacing and plant height are considered in our solution. We will extend to new phenotyping parameters, such as leaf number, leaf angle, and leaf length. Sixth, we hope to employ machine learning or deep learning in the future, to enhance the speed and accuracy performances of our solution. For example, fast and robust principal component analysis (Sun and Du, 2018) and joint sparse representation-based classification (Peng et al., 2019) can be used for organ detection and terrain-plant classification. We also expect that negative effects due to wind-induced deformations can be decreased by learning algorithms.

## Author Contributions

QQ, NS, NW, ZF, and YW designed the data collection experiments. NS and ZF carried out the data collection experiments. QQ, NS, HB, NW, and BL designed the data processing strategy. QQ and NS developed the data processing codes. QQ, NS, and HB analyzed the data processing results. QQ, ZF, ZM, and YC developed the mobile robot. QQ, NS, and BL wrote the first version of the manuscript. QQ, HB, NW, and ZM discussed the data analysis results and revised the manuscript.

## Conflict of Interest Statement

The authors declare that the research was conducted in the absence of any commercial or financial relationships that could be construed as a potential conflict of interest.

## Footnotes

1 https://github.com/ros-drivers/velodyne

2 velodyne/velodyne_driver/src/driver/driver.cc
